# Cerebral blood flow regulation and cognitive function: a role of arterial baroreflex function

**DOI:** 10.1007/s12576-019-00704-6

**Published:** 2019-08-23

**Authors:** Shigehiko Ogoh, Takashi Tarumi

**Affiliations:** 1 Department of Biomedical Engineering, Toyo University, 2100 Kujirai, Kawagoe, Saitama 350-8585 Japan; 2 Human Informatics Research Institute, National Institute of Advanced Industrial Science and Technology, Tsukuba, Japan

**Keywords:** Cerebral autoregulation, Cerebral CO_2_ reactivity, CBF regulation, Cardioplumonary baroreflex, Autonomic function, Systemic blood pressure regulation

## Abstract

A strict adequate perfusion pressure via arterial baroreflex for the delivery of oxygen to the tissues of the body is well established; however, the importance of baroreflex for cerebral blood flow (CBF) is unclear. On the other hand, there is convincing evidence for arterial baroreflex function playing an important role in maintaining brain homeostasis, e.g., cerebral metabolism, cerebral hemodynamics, and cognitive function. For example, mild cognitive impairment attenuates the sensitivity of baroreflex, and Alzheimer’s disease further decreases it. These clinical findings suggest that CBF and cerebral function are affected by systemic blood pressure regulation via the arterial baroreflex. However, dysfunction of arterial baroreflex is likely to affect CBF regulation as well as the underlying neuronal function, but identifying how this is achieved is arduous since neurological diseases affect systemic as well as cerebral circulation independently. Recent insights into the influence of blood pressure regulation via the arterial baroreflex on cerebral function and blood flow regulation may help elucidate this important question. This review summarizes some update findings regarding direct (autonomic regulation) and indirect (systemic blood pressure regulation) contributions of the arterial baroreflex to the maintenance of cerebral vasculature regulation.

## Introduction

**Fig. 1:**
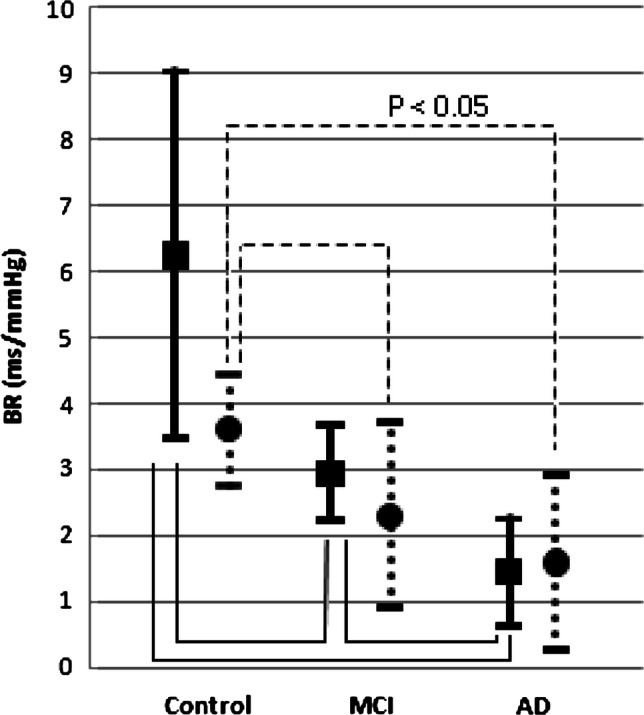
Cognitive impairment and baroreflex. Mild cognitive impairment (MCI) attenuates baroreflex sensitivity (BR), and BR is further reduced in Alzheimer’s disease (AD), indicating that cognitive function may be associated with arterial baroreflex function [5]. The solid lines represent the derivation sample and the dashed lines represent the validation sample. BR obtained by the ARXAR model

An adequate regulation of arterial blood pressure (ABP) is attained by the arterial baroreflex modulation of sympathetic outflow to the heart and vasculature, and parasympathetic nerve activity to the heart [1–4]. On the other hand, the importance of baroreflex for cerebral blood flow (CBF) remains unclear because the effect of change in ABP on CBF is dampened by cerebral autoregulation. However, there is convincing evidence for arterial baroreflex function playing an important role in maintaining brain homeostasis, e.g., cerebral metabolism, cerebral hemodynamics, and cognitive function [5, 6]. For example, mild cognitive impairment (MCI) attenuates the sensitivity of baroreflex, and Alzheimer’s disease further decreases it (Fig. 1) [5]. In addition, Tarumi et al. [6] compared healthy subjects to patients with MCI and demonstrated that cerebral neuronal fiber integrity is associated with arterial baroreflex function and cognitive performance. By contrast, a recent study demonstrated that CBF regulation, e.g., dynamic cerebral autoregulation was not altered in patients with dementia despite a decrease in CBF [7]. Therefore, these clinical findings suggest an important role of blood pressure regulation for maintaining brain homeostasis because cognitive function are likely affected by systemic blood pressure regulation via arterial baroreflex rather than cerebral vascular regulation.

Although hypertension is recognized as the established risk factor for cerebrovascular disease, the “selfish brain hypothesis” conceived by Dickinson and Thomason [8] several years ago suggested that high blood pressure is necessary for the maintenance of cerebral perfusion. On the basis of this hypothesis, hypertension could be thought as a consequence of cerebral hypoperfusion, especially in the posterior cerebral artery that supplies blood to the brainstem (center of the nervous system). This concept may be reasonable because cerebrovascular remodeling and hypoperfusion occur prior to the development of hypertension [9]. Moreover, while treated hypertensive patients have normal blood pressure, their CBF and perfusion are lower with increased cerebrovascular resistance when compared with non-treated hypertensive patients [10]. Cerebral hyperperfusion may enhance the risk of damaging the blood–brain barrier whereas cerebral hypoperfusion may attenuate brain function, including the autonomic nervous system. Indeed, the risk of dementia development increases in patients with hypertension treated with antihypertensive drugs [11]. Thus, the control of cerebral perfusion is important for maintaining an adequate neuronal micro-environmental homeostasis as well as autonomic function, indicating that CBF regulation is tightly linked to blood pressure regulation.

Previous studies provided a possible clue that the arterial baroreflex could mediate the relation between CBF regulation and systemic blood pressure. However, CBF regulation is highly complex and influenced by neurogenic, hemodynamic, autoregulatory, and metabolic factors. This implies that precise direct (autonomic regulation) and indirect (systemic blood pressure regulation) contributions of the arterial baroreflex to the maintenance of cerebral vasculature regulation are challenging to distinguish. Nevertheless, recent insights into the influence of blood pressure regulation via the arterial baroreflex on cerebral function and blood flow regulation may help elucidate this conflict.

## Baroreflex and CBF regulation

### Arterial baroreflex-systemic blood pressure regulation

Alterations in ABP cause a conformational change in the baroreceptors, located in the carotid sinus bifurcation and aortic arch, leading to changes in afferent neuronal firing. A branch of the glossopharyngeal nerve, the Hering nerve, carries impulses from the carotid baroreceptors, and small vagal branches carry impulses from the aortic baroreceptors. These afferent signals converge centrally within the nucleus tractus solitarii (NTS) of the medulla oblongata. The carotid mechanoreceptors function as the sensors in a negative feedback control system [12]. The neural adjustments will affect both the heart and the blood vessels in an appropriate fashion to allow ABP to regain its original pressure. The baroreflex-mediated modifications of autonomic nervous activity and ABP may influence cerebral vasculature, although their relative contributions remain unclear. For example, acute hypertension decreases sympathetic nerve activity via the arterial baroreflex resulting in peripheral vasodilation. In contrast, the constriction of the cerebral vasculature is required for protecting the blood–brain barrier against acute hypertension via autoregulation. However, we currently do not know whether baroreflex-induced decrease in sympathetic nerve activity modifies cerebral vasoconstriction elicited by autoregulation.

### CBF regulation

#### Cerebral autoregulation

The classic work of Lassen [13] established the concept that human CBF is maintained within a narrow range despite changes in mean arterial pressure between 60 and 150 mmHg. This relationship, termed cerebral autoregulation (CA), is an established homeostatic mechanism of blood flow regulation in the brain that buffers fluctuations in CBF when cerebral perfusion pressure changes and acts through vasomotor effectors that control cerebral vascular resistance [14]. An acute increase in cerebral perfusion pressure causes cerebral vasoconstriction, and conversely, an acute decrease in cerebral perfusion pressure causes cerebral vasodilation to maintain CBF relatively constant within the range of CA between 60 and 150 mmHg [13].

#### Partial pressure of arterial carbon dioxide (PaCO_2_)

Change in PaCO_2_, a powerful mediator of CBF, induces a response in the CBF termed cerebrovascular CO_2_ reactivity. Hypocapnia causes cerebral vasoconstriction and reduces CBF, which attenuates the further decrease in brain tissue PCO_2_. By contrast, hypercapnia increases CBF through cerebral vasodilation, thereby limiting elevations in brain tissue PCO_2_. The level of cerebral neural activation, such as that occurs during sleep, influences cerebrovascular reactivity to CO_2_ [15]. Dynamic CA, which is the rapid change in CBF that buffers a transient change in ABP, is influenced by cerebrovascular reactivity to CO_2_ [16] because hypotension or hypertension causes CO_2_ accumulation (hypercapnia) or CO_2_ washout (hypocapnia), respectively.

#### The interaction between dynamic CA and cerebrovascular CO_2_ reactivity

In the past, CA was mainly evaluated by the steady-state relationship between CBF and blood pressure [13]. This method is termed as “static” autoregulation testing [17]. Also, CA can be evaluated by dynamic approach using measurement of relative CBF changes in response to a rapid change in blood pressure. Based on the different methodologies, CA evaluated by the dynamic approach termed “dynamic CA” which is distinguished with “static CA” evaluated by static autoregulation testing [17]. The early work of Aaslid et al. [14], using dynamic approach, provided experimental evidence that hypocapnia improves dynamic CA whereas hypercapnia impairs it. Since cerebrovascular CO_2_ reactivity is tightly linked to the ventilatory response to CO_2_, CBF regulation is affected by central chemoreflex control of minute ventilation (*V*_E_). Of note, an abnormal chemoreflex control of breathing is evident in a range of pathological conditions (e.g., chronic lung disease, heart failure, and sleep apnea) and may alter dynamic CBF regulation [18–20]. However, the physiological significance or impact of this alteration in dynamic CA via changes in arterial CO_2_ remains unknown. Physiologically, it is possible to speculate that the attenuation in CBF regulation under hypercapnia or hypoxia compensates for abnormal gas concentrations in the brain. For example, dynamic CA is attenuated by hypercapnia caused by central respiratory chemoreflex dysfunction, which accelerates an increase in CBF as the blood pressure rises. This additional increase in CBF enhances CO_2_ washout because of hypercapnia and consequently reduces acidosis associated with hypercapnia. The attenuation in dynamic CA may be necessary for countering a dysfunctional central respiratory chemoreflex to maintain CO_2_ homeostasis.

### Paradox of baroreflex function and CBF regulation: dynamic CA

**Fig. 2:**
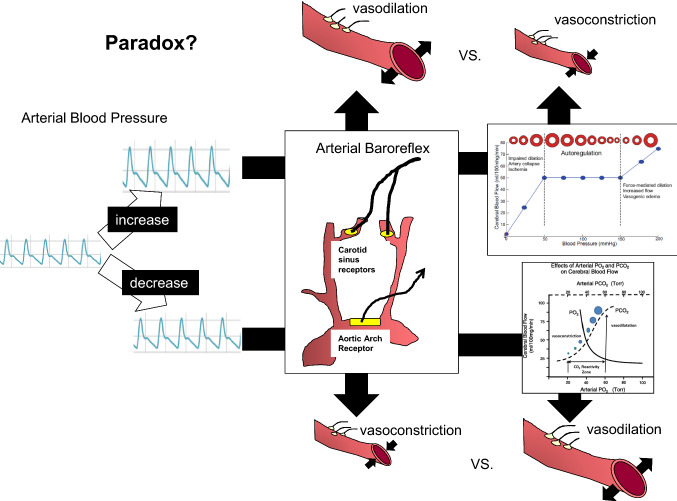
Paradox of CBF regulation and baroreflex. An acute increase in arterial blood pressure (ABP) causes cerebral vasodilation in the context of arterial baroreflex control of autonomic activity. By contrast, an acute increase in ABP causes cerebral vasoconstriction to protect the blood–brain barrier via cerebral autoregulation. On the other hand, an acute decrease in ABP causes cerebral vasoconstriction in the context of arterial baroreflex control of autonomic activity, and it causes cerebral vasodilation to washout carbon dioxide (CO_2_) in the brain via cerebrovascular CO_2_ reactivity

The dynamic CA and arterial baroreflex function via the autonomic nervous system have a paradoxical effect on CBF regulation. For example, during an acute increase in ABP, loading of arterial baroreceptors inhibits sympathetic outflow while enhancing vagal activity at the NTS of the medulla oblongata, and consequently decreases ABP to around baseline levels with reduction in HR and peripheral vascular resistance [1–3, 12]. Importantly, if sympathetic nerve activation directly affects cerebral vasculature, baroreceptor loading can induce the reduction of sympathetic nerve activity which may subsequently dilate the cerebral vasculature similar to peripheral vasculature. By contrast, an acute increase in ABP causes cerebral vasoconstriction via dynamic CA to maintain CBF relatively constant [13]. In other words, these different physiological mechanisms cause a paradox in cerebral vascular control; an acute increase in ABP may induce cerebral vasodilation via arterial baroreflex control of autonomic nerve system while dynamic CA constricts the cerebral vasculature (Fig. 2). Under this background, it is a possibility that there is a regional difference in autonomic outflow between systemic and cerebral blood vessels; however, a role of sympathetic nerve activity on cerebral vasculature remains controversial in humans.

### Paradox of baroreflex function and CBF regulation: cerebrovascular CO_2_ reactivity

Similarly, the cerebrovascular reactivity to CO_2_ may not correlate to the vascular response to a change in autonomic activity. For example, as opposed to the increased ABP described earlier, an acute decrease in ABP causes unloading of arterial baroreceptors, which activates sympathetic outflow and inhibits vagal activity in the cardiovascular centers, and consequently increases ABP to around baseline levels with augmentations of HR and peripheral vascular resistance [1–3, 12]. If autonomic control of cerebral vasculature is similar to peripheral vasculature, an increase in sympathetic nerve activity via unloading of baroreceptors may cause cerebral vasoconstriction during decrease in ABP. However, an acute decrease in ABP prevents CO_2_ wash-out from the brain tissue, and consequently cerebrovascular CO_2_ reactivity causes cerebral vasodilation for the prevention of acidosis in the brain. Taken together, an acute decrease in ABP constricts cerebral vasculature via arterial baroreflex control of autonomic nervous system whereas cerebrovascular CO_2_ reactivity causes cerebral vasodilation (Fig. 2). Thus, cerebral vasomotion via the baroreflex may be viewed as a paradoxical reaction with little physiological benefit [21]. This inconsistency between the arterial baroreflex and dynamic CA or cerebrovascular CO_2_ reactivity regarding cerebral vasculature lacks explanation and complicates the actual role of arterial baroreflex on CBF regulation. It is plausible that the response of the cerebral vasculature to autonomic activity may differ from that of the peripheral vasculature.

**Fig. 3:**
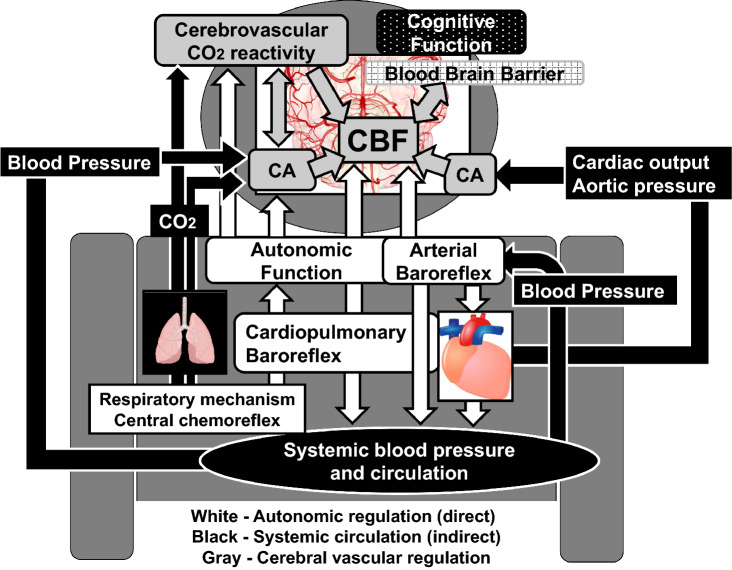
The schematic of direct and indirect effects of barorefex on cerebral vasculature. Cerebral vasculature is affected by autonomic nervous system via baroreflex and other physiological mechanisms (direct effect, white). On the other hand, baroreflex control of autonomic function regulates systemic circulation which affects cerebral vasculature (indirect effect, black). Moreover, cerebral autoregulation (CA) and cerebrovascular CO_2_ reactivity regulates cerebral blood flow (CBF) (gray), and both CBF regulatory system are affected by autonomic function and systemic circulation

In the following two paragraphs, we discuss direct and indirect effects of arterial baroreflex on cerebral vasculature. Summarized schematic is presented in Fig. 3.

## Direct effect of arterial baroreflex (autonomic regulation) on cerebral vasculature

A direct association between the arterial baroreflex and cerebral circulation, via the autonomic nervous system, has been demonstrated in some animal models [22–26]. For example, in rats with cervical cordotomy and vagotomy, regional CBF (the frontal and occipital cortices) was increased by eclectically stimulating the intermediate portion of the solitary nucleus [25]. On the other hand, sinoaortic denervation eliminates cerebral vasodilatation during acute hypertension [27]. In addition, cerebral vasomotion was induced by baroreceptor stimulation [27–29] while sympathetic efferent to the cervical sympathetic trunk was elevated during baroreflex deactivation [29]. Similarly, CBF was increased by chemical stimulation of the ventrolateral medullary depressor area in anesthetized rats [30–32]. Moreover, lesions of nucleus tractus solitarii impaired CBF regulation (e.g., CA) [23]. These previous animal studies partly explain the effect of baroreflex-induced change in autonomic activity on cerebral vasculature.

However, these previous animal studies demonstrated the only open-loop characteristic of autonomic neural effect on CBF (under anesthesia, etc.) although baroreflex activation can alter many physiological factors that can affect cerebral vasculature (Fig. 3). Therefore, the role of autonomic control via the arterial baroreflex on CBF regulation remains unclear. Indeed, traditionally in humans, increases in sympathetic activity have a limited effect on the cerebral vasculature, particularly at rest. Indeed, the effect of the cervical sympathetic stimulation on retinal oxygen tension and on uveal, retinal, and CBF was minimal, although the cerebral vasculature is innervated with sympathetic nerve fibers in humans [33, 34].

Interestingly, Heistad et al. [35] demonstrated that sympathetic stimulation decreases CBF during severe hypertension in animal model despite its minimal response under resting conditions. These findings from animals can be translated into humans such that inhibition of sympathetic activation using prazosin (an α-1 adrenergic receptor blocker) did not alter CBF under resting conditions in normotensive humans [36], but it increased CBF in hypertensive patients along with reductions in blood pressure [37]. In addition, high-intensity exercise (10-repetition maximum leg press exercise) has been shown to elicit increase in cerebral vascular resistance [38]. The CBF responses to sympathoexcitation seen in previous studies have reasonable physiological impacts because cerebral vasoconstriction may be an important mechanism to prevent regional over-perfusion and damage to the blood–brain barrier against hypertension. Moreover, it has been well established that autonomic nervous activity modifies the mechanism of CBF regulation. The blockade of sympathetic activation enhances the reactivity of CBF to PaCO_2_ [39] and attenuates CA [40, 41].

Importantly, these findings suggest that a different physiological condition, particularly under hypertensive conditions, modifies or enhances the direct effect of change in sympathetic nervous activity on cerebral vasculature [33, 35–37] and CBF regulatory mechanisms [39–41]. In other words, the contribution of sympathetic nervous activity to CBF regulation depends on physiological and pathological conditions, which is perhaps why the direct effect of sympathetic nerve activity on CBF regulation, established by others, remains inconsistent. It is plausible that this heterogenous contribution of autonomic function to cerebral vasculature may limit regional over-perfusion and protect against the breakdown of the blood–brain barrier [35, 37, 40, 42], however, these possibilities have not been elucidated clearly. For example, previous studies [21, 43, 44] reported that CBF decreases during orthostatic stress which suggests that such cerebral vascular response is likely induced by baroreflex-mediated sympathetic activation. However, this CBF response is difficult to understand physiologically since arterial and cardiopulmonary baroreflexes are the major mechanisms for maintaining perfusion pressure and neuronal homeostasis for the brain. Zhang et al. [45] using autonomic ganglionic blockade, confirmed this question and demonstrated that orthostatic-stress-induced sympathoexcitation via baroreflexes did not affect cerebral vasculature. Therefore, in this condition, the major influence of the arterial baroreflex on CBF regulation may be an indirect result of its hemodynamic effects.

## Indirect effect of arterial baroreflex (systemic blood pressure regulation) on cerebral vasculature

### Does cardiac output affect CBF?

**Fig. 4:**
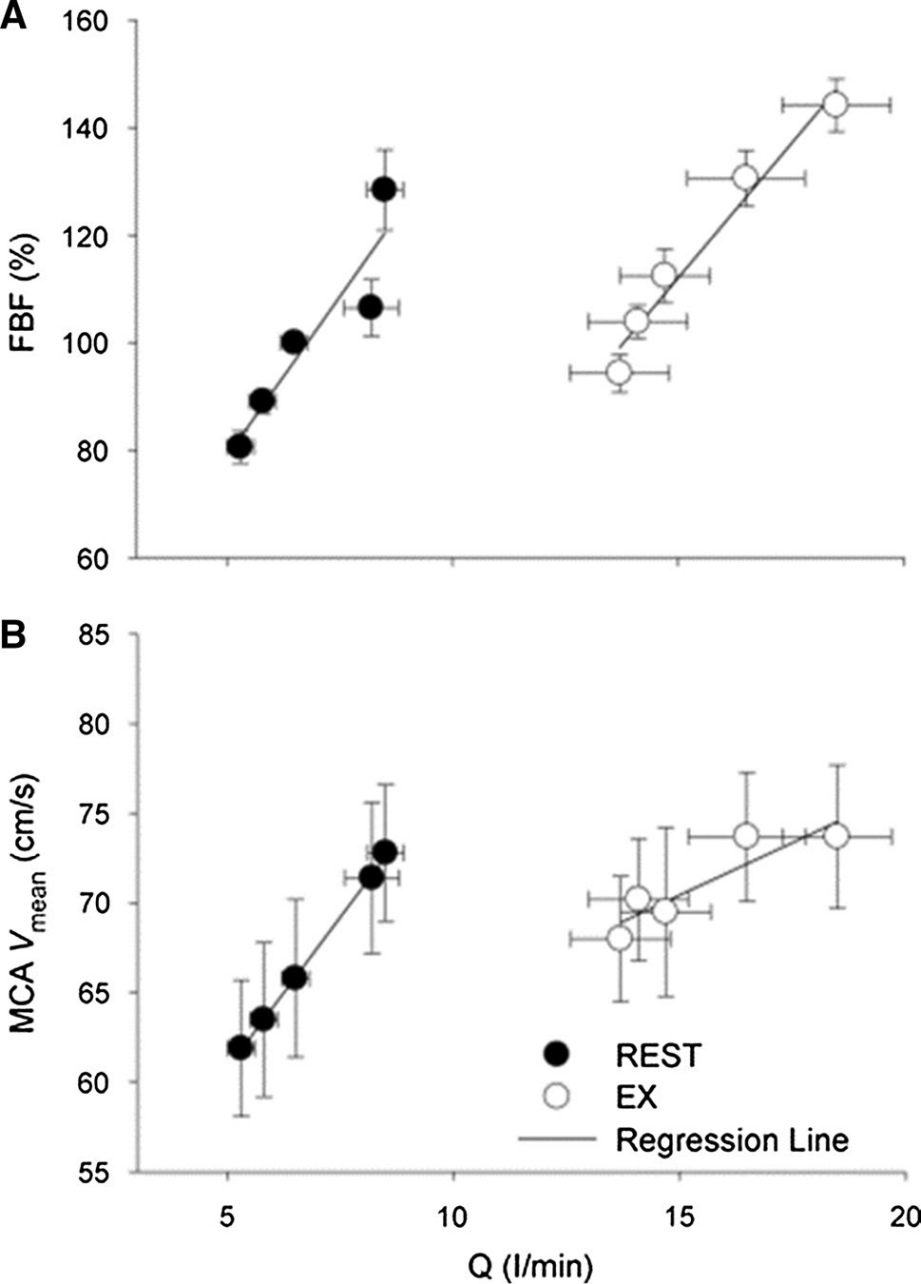
The relationship between CBF and cardiac output (*Q*). Middle cerebral artery mean blood velocity (MCA *V*_mean_) as an index of cerebral blood flow as well as forearm blood flow (FBF) was linearly related to the change in cardiac output (*Q*) at rest and during exercise [49]

Cardiac output likely influences CBF directly since the normal increase in CBF during dynamic exercise is reduced when the cardiac output is attenuated by β_1_-blockade [46] or in patients with heart failure [47] or atrial fibrillation [48]. We observed that CBF was linearly related to the change in cardiac output at rest and during exercise (Fig. 4) [49]. Furthermore, the slope of the linear regression between the cardiac output and CBF was greater at rest (*P* = 0.035) than during exercise [49]. The contribution of the changes in cardiac output to the carotid-baroreflex control of ABP during exercise was found to be minimal, even though the carotid-baroreflex-mediated change in total vascular conductance contributed significantly to the change in ABP [5, 39, 40]. These findings indicated that cardiac output was an important determinant of the CBF especially during exercise, and any changes in cardiac output via the arterial baroreflex could directly influence CBF [50]. Nevertheless, on the basis of our previous study [51], the carotid-baroreflex control of the heart may provide an under-appreciated regulation of CBF.

### Does cardiac baroreflex function contribute to dynamic CA?

**Fig. 5:**
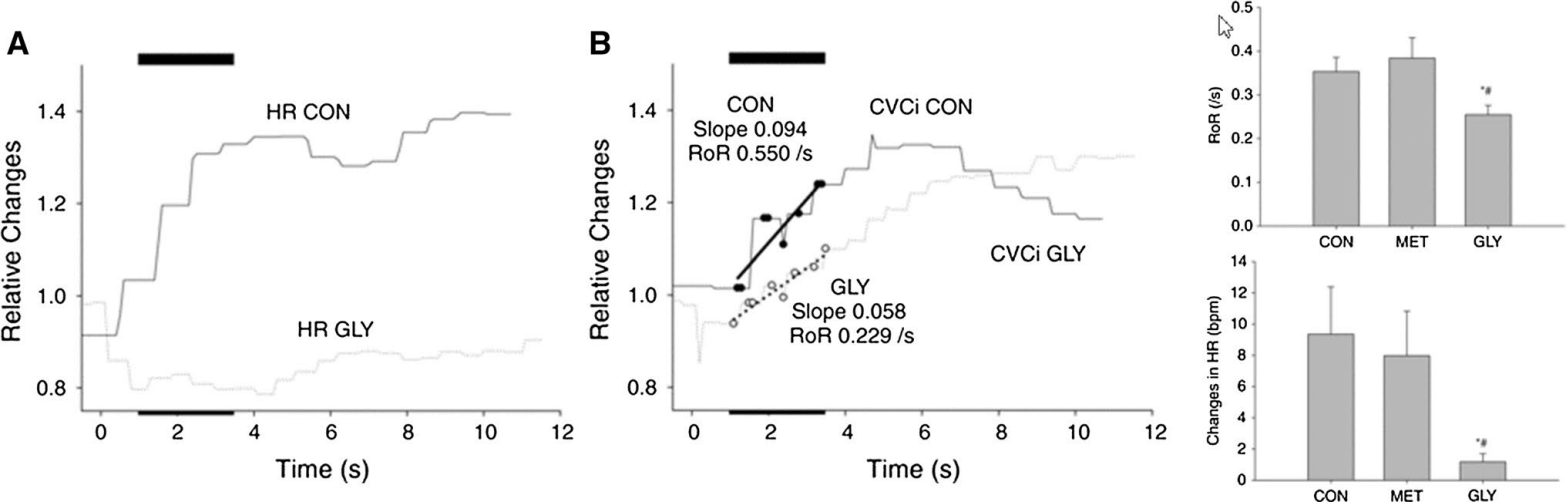
The role of cardiac baroreflex function for dynamic cerebral autoregulation. Thigh cuff release elicited a transient drop in arterial blood pressure and resultant tachycardia via the arterial baroreflex (**a**) and increase in cerebral vascular conductance (CVCi) via cerebral autoregulation (**b**). Vagal blockade (glycopyrrolate, GLY) attenuated this tachycardic response, and the rate of regulation (RoR) as an index of dynamic cerebral autoregulation was also inhibited in the vagal blockade condition compared to both control (CON) and β_1_-adrenergic blockade (metoprolol, MET) conditions, indicating that the baroreflex-induced tachycardia response plays an important role in dynamic cerebral blood flow regulation [51]

Thigh cuff release has often been applied for the evaluation of dynamic CA because it is a non-invasive and non-pharmacological method [17, 52]. However, thigh cuff release elicits an integrated physiological response that involves not only cerebrovascular changes but also arterial baroreflex-mediated changes in peripheral vasculature and HR [53]. As the drop in arterial pressure associated with thigh cuff release does not alter central venous pressure (and hence SV) [54], the reflex tachycardia transiently augments the cardiac output in proportion to the degree of tachycardia. In this context, we have identified dynamic CA during acute hypotension with and without arterial baroreflex-mediated tachycardia and the consequent changes in cardiac output [51]. Hypotension was induced before and after sympathetic or cholinergic blockade. Thigh cuff release elicited a transient drop in ABP and the resultant tachycardia, which diminished with vagal blockade. Dynamic CA was also attenuated in the vagal blockade condition compared to both control and β_1_-adrenergic blockade conditions and was related to the attenuated tachycardia response (Fig. 5). These data also highlight the important role of the cardiac baroreflex in CBF regulation.

### Cardiac function directly contributes to CBF

**Fig. 6:**
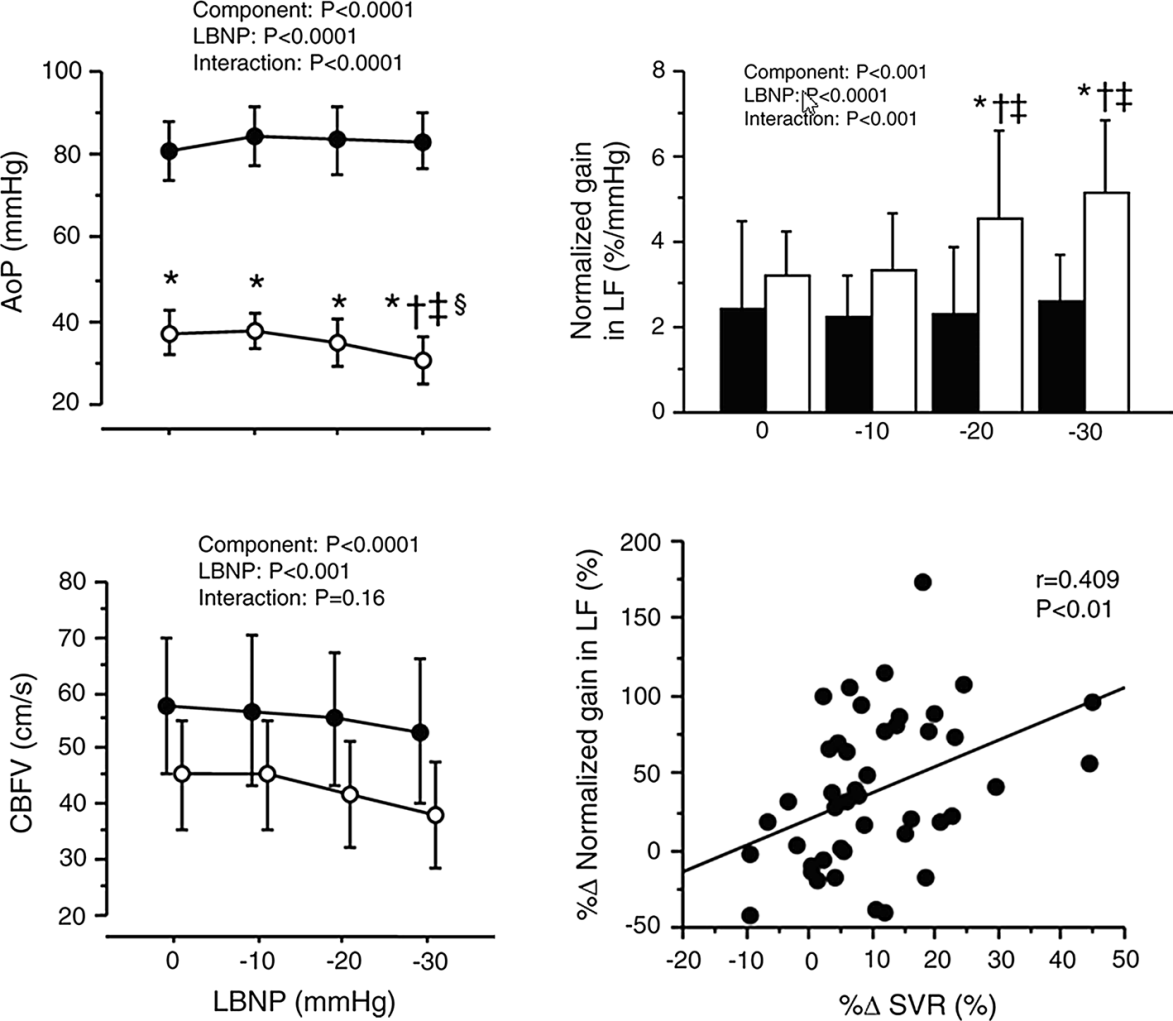
The transition from aortic pressure (AoP) to CBF. Lower body negative pressure (LBNP) during orthostatic stress decreased cerebral blood flow velocity (CBFV) as well as AoP, and the gain of dynamic transition from pulsatile AoP to CBFV increased gradually during a graded LBNP. Moreover, this gain is associated with an arterial baroreflex-induced change in systemic vascular resistance (SVR). The closed circles or bars represent the steady-state component of hemodynamics, and the open circles or bars indicate a pulsatile component of hemodynamics [55]

Since orthostatic stress (e.g., lower body negative pressure (LBNP)) decreases central blood volume and consequently reduces CBF, cardiac function thereby affects CBF. In our previous study [55], we characterized the pulsatile hemodynamic transmission from the heart to the brain by frequency-domain analysis (Fig. 6). Aortic pressure (AoP) was well maintain but pulsatility of AoP gradually decreased during LBNP. Novel findings of this study are that the normalized transfer function gain from pulsatile AoP to pulsatile CBF velocity is significantly augmented with a mild LBNP stimulation (− 20 and − 30 mmHg of LBNP, Fig. 6, right upper) and that such response is associated with the increase in systemic vascular resistance via an unloading of arterial baroreceptors (Fig. 6, right bottom). In the other words, baroreflex-induced systemic vasoconstriction may deteriorate the dampening effect on slow oscillations of pulsatile hemodynamics toward the brain. However, these findings also provide the evidence that baroreflex function affects CBF regulation indirectly.

### Peripheral vascular response via arterial and cardiopulmonary baroreflex may contribute to CBF

**Fig. 7:**
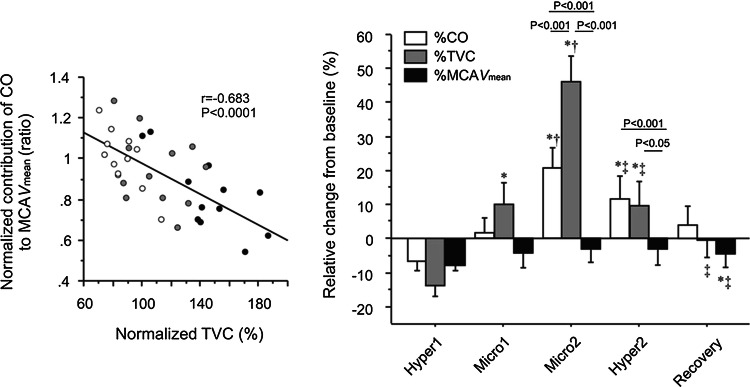
Effect of cute hypervolemia on CBF and peripheral vasculature via baroreflex. During parabolic flight, acute loading of baroreceptors drastically evokes a decrease in sympathetic nerve activity and consequently reduces peripheral vascular resistance, and the middle cerebral artery mean blood velocity (MCA *V*_mean_) remains unchanged. This change in total vascular conductance (TVC) via the arterial baroreflex is associated with a change in the contribution of the cardiac output (CO) to MCA *V*_mean_. *Hyper* hypergravity, *micro* microgravity [56]

Compared with a central hypovolemia condition such as LBNP, the hypervolemia induced by microgravity maintained adequate CBF via the arterial and cardiopulmonary baroreflex [56]. During parabolic flight, acute loading of baroreceptors drastically evoked a decrease in sympathetic nerve activity and consequently reduced peripheral vascular resistance independently from the cerebral vasculature (Fig. 7). In addition, an acute hypervolemia attenuated dynamic CA [57]. These findings demonstrated that baroreflex function mainly affects CBF regulation via the controlled peripheral vasculature independently from the direct influence of the autonomic nervous system. This regulatory mechanism should be important in maintaining adequate CBF during a hypervolemia condition. If the peripheral vasculature is not controlled by a change in central blood volume and blood pressure during a hypervolemia condition, cerebral hyperperfusion may damage the blood–brain barrier. In addition, if the baroreflex-mediated autonomic control affects CBF directly, then it is not well controlled adequately because a hypervolemia-induced decrease in sympathetic nerve activity causes vasodilation, indicating more increase in CBF.

### Baroreflex function is associated with cerebral neuronal fiber integrity

**Fig. 8:**
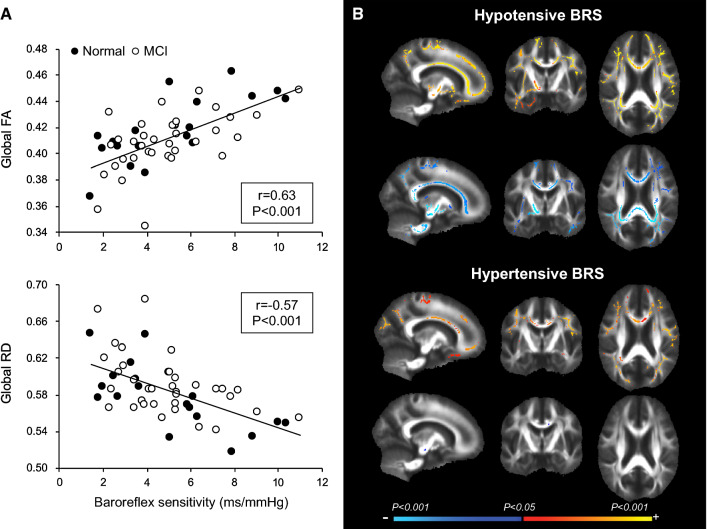
The relationship between baroreflex sensitivity and brain white matter neural fiber integrity. **a** Reduced baroreflex sensitivity is associated with lower fractional anisotropy (FA) and higher radial diffusivity (RD) in older adults, regardless of their cognitive status. FA provides a summary measure of axonal fiber tract integrity whereas RD reflects axonal demyelination. **b** BRS calculated during hypotension (top) showed a stronger relation with FA and RD than that during hypertension (bottom). Color bar shows the directionality of correlation and the magnitude of *P* value. The color in brain maps represent the statistically significant areas that are associated with BRS [6]. *MCI* mild cognitive impairment (color figure online)

Baroreflex sensitivity has been shown to correlate with cerebral neuronal fiber integrity and aortic stiffness [6]. Our recent study tested the elderly subjects with normal cognitive function and amnestic MCI, a prodromal phase of Alzheimer’s disease (Fig. 8). Cardiovagal baroreflex sensitivity was assessed using the modified Oxford method in which intravenous bolus injections of sodium nitroprusside, followed by phenylephrine, were delivered to induce a large change in ABP [58]. On a separate visit, the participants underwent brain MRI to assess their white matter axonal fiber tract integrity via diffusion tensor imaging, which quantifies the directionality and magnitude of water diffusion inside the axonal fiber tracts and provides microstructural integrity measures of the cerebral white matter [59]. This study demonstrated that lower fractional anisotropy (FA) and higher radial diffusivity (RD) is associated with reduced baroreflex sensitivity and worse executive function performance in older adults, regardless of their cognitive status. Physiologically, lower FA reflects an overall reduction of neuronal fiber tract integrity, whereas higher RD is suggestive of axonal demyelination [59, 60]. Furthermore, when analyzing the hypotensive and hypertensive episodes separately during the modified Oxford maneuver, we observed a stronger association of neuronal fiber integrity with baroreflex sensitivity during hypotension compared with hypertension. Therefore, these results collectively suggested that impaired baroreflex-mediated ABP regulation, especially during hypotension, may negatively impact brain structures and also serve as a cardiovascular marker for detecting aged individuals at increased risk for cognitive impairment [5].

## Summary

Evidence from previous studies indicated that there are both direct and indirect effects of arterial baroreflex on CBF regulation (Fig. 3). Therefore, there is no doubt that the arterial baroreflex plays an important role in the regulation of CBF, likely by controlling systemic circulation. However, arterial baroreflex control of CBF is not exactly same as the peripheral vasculature because cerebral circulation is not only under the systemic effects of autonomic neural function but also the unique mechanisms of CA and cerebrovascular CO_2_ reactivity in the closed loop condition. Therefore, the relative contribution of autonomic function to CBF regulation remains unknown, and the physiological role of baroreflex for CBF regulation also remains controversial. For example, when system ABP increases acutely, baroreflex causes peripheral vasodilation to reduce ABP, but cerebral vasculature needs to be constricted to protect blood brain barrier. Indeed, acute hypertension does not decrease CBF or cause cerebral vasodilation by baroreceptor loading. It is possible that there is a regional difference in autonomic outflow between systemic and cerebral blood vessels. Moreover, the contribution of direct (autonomic regulation) and indirect (systemic blood pressure regulation) baroreflex influences on CBF regulation may not be steady. For example, in patients with hypertension, direct sympathetic nerve activation causes cerebral vasoconstriction but it does not occur in normotensive subjects. The complexity of the relationship between the arterial baroreflex and many of the other mechanisms intricately involved in the regulation of CBF (e.g., cardiac output, PaCO_2_, PaO_2_, and respiratory chemoreflex) is an important consideration. Moreover, it is known that sympathetic nerve activation as well as cardiac output and respiratory system modifies the CBF regulatory system (e.g., CA and cerebrovascular CO_2_ reactivity). Therefore, alterations in the arterial baroreflex or other physiological factors to the maintenance of adequate CBF particularly in disease conditions, e.g., cardiovascular disease, should be considered, although a role for arterial baroreflex in CBF control has been challenging to identify and thus underappreciated.
